# Transcriptional profiling reveals differentially expressed genes involved in lipid biosynthesis during cacao seed development

**DOI:** 10.1038/s41598-019-53959-9

**Published:** 2019-11-21

**Authors:** Fupeng Li, Baoduo Wu, Lin Yan, Chaoyun Hao, Xiaowei Qin, Jianxiong Lai, Yinghui Song

**Affiliations:** Spice and Beverage Research Institute, Chinese Academy of Tropical Agricultural Sciences/Key Laboratory of Genetic Resources Utilization of Spice and Beverage Crops, Ministry of Agriculture, Wanning, 571533 P.R. China

**Keywords:** Transcriptomics, Plant molecular biology

## Abstract

*Theobroma cacao* is a plant of economic value due to the use of its seed lipid for chocolate, confectionery, and cosmetic industries. The seed lipid contains a stable ratio of saturated and unsaturated fatty acids, which determines its unique melting temperature. However, little is known about the molecular mechanism determining the fatty acid ratio and lipid content in cacao. To gain insight into the unique properties of lipid synthesis in cacao, biochemical and transcriptomic approaches were used to compare the lipid accumulation between high and low lipid content cacao accessions. Lipid accumulation rates and lipid content were different between the two accessions. Moreover, differentially expressed genes were detected between high and low lipid content cacao accessions. The data allowed the identification of distinct candidate genes and furthered our understanding of lipid accumulation, potentially explaining the differences in lipid content between various cacao accessions. The results might be used to develop molecular tools and engineer alternative pathways for cacao breeding with improved lipid production potentials.

## Introduction

Cacao (*Theobroma cacao* L.) is a neotropical species native to Amazonian lowland rainforests and is now grown in more than 50 countries for the livelihoods of 40 to 50 million people worldwide^1,2^. The main producers of cacao are Cote d’Ivoire (32.2%), Ghana (19.3%), Indonesia (16.4%), Brazil (6.2%), Cameroon (6.1%) and Nigeria (5.6%), and both marketing and consumption involves many countries around the world^3^. Cacao beans, containing approximately 50% total lipid (cocoa butter), are used for the chocolate, confectionery, and cosmetic industries^4^. Cocoa butter is mainly composed of palmitic acid (C16:0), stearic acid (C18:0), oleic acid (C18:1), and other fatty acids, which organize as symmetrical triglycerides (TAGs) of POP, POS and SOS^5^. The ratios of POP, POS and SOS have been shown in conventional cocoa butter to be 22, 46, and 32, the composition of which determines its unique melting temperature at 36–37 °C^6,7^.

Cacao pods appear as tiny cucumber-like projections emerging from small flowers and grow rapidly in the earliest stage. When pods are 15 to 17 weeks old, the ovules begin to continuously solidify from unsolidified gel-like material until the seeds visually appear to be solid. As the pods develop over the last 8 weeks, the seeds enlarge and deepen in colour until they are violet^8^. Lipid accumulation proceeds with seed maturation. During the early stage of seed development, a major shift occurs in fatty acid composition between 104 and 125 days after pollination (DAP) when the transiently high proportion (18%) of linoleic acid (C18:2) rapidly drops to approximately 3%. From this shift onward, a normal fatty acid composition is quickly established and does not change materially thereafter^9,10^.

In plants, *de novo* synthesis of fatty acids occurs with the conversion of sucrose into acetyl coenzyme A (acetyl-CoA), an intermediary in several metabolic pathways that can also be metabolized from hexose phosphate or pyruvate^11,12^. The formation of malonyl-CoA from acetyl-CoA by acetyl-CoA carboxylase (ACC) is a key regulatory step of the fatty acid biosynthetic process. Subsequently, the malonyl group is transferred from CoA to a protein cofactor named acyl carrier protein (ACP) by a malonyl-CoA:acyl carrier protein S-malonytransferase (MAT)^13^. Given the function of fatty acid synthase (FAS) in fatty acid synthesis, fatty acid chains are continuously elongated in two-carbon increments, producing palmitoyl-ACP (16:0-ACP) and stearoyl-ACP (18:0-ACP)^14^. Some 16:0-ACP is released from the FAS machinery before its conversion to 18:0-ACP, thus resulting in the export of palmitate (C16:0) for triacylglycerol synthesis. In most species, only limited amounts of stearate (C18:0) are exported from the plastids and accumulated in seeds^12^. Molecules of 18:0-ACP are efficiently desaturated by stearoyl-ACP desaturase (SAD), resulting in the conversion of 18:0-ACP into 18:1-ACP and producing oleate (C18:1). Triacylglycerol (TAG) assembly utilizes G3P and acyl-CoAs as primary substrates for glycerol-3-phosphate acyltransferase (GPAT), lysophosphatidic acid acyltransferase (LPAT), and diacylglycerol acyltransferase (DGAT)^12^.

The fatty acid biosynthetic pathway and lipid accumulation in cacao has been poorly studied in previous investigations, despite the uniqueness of cocoa butter for making chocolate. In this study, to obtain an overall comprehensive characterization of fatty acids and lipid metabolism in the cacao seed, lipid temporal accumulation patterns, transcriptional analysis, and differential gene expression profiles were assessed. The unique characteristics of lipid metabolism were described, revealing insight into transcriptional coordination in the cacao seed. The results generated in the present study provide an important foundation to further explore the regulatory mechanism of cacao seed lipid accumulation.

## Results

### Seed development and lipid content

**Figure 1:**
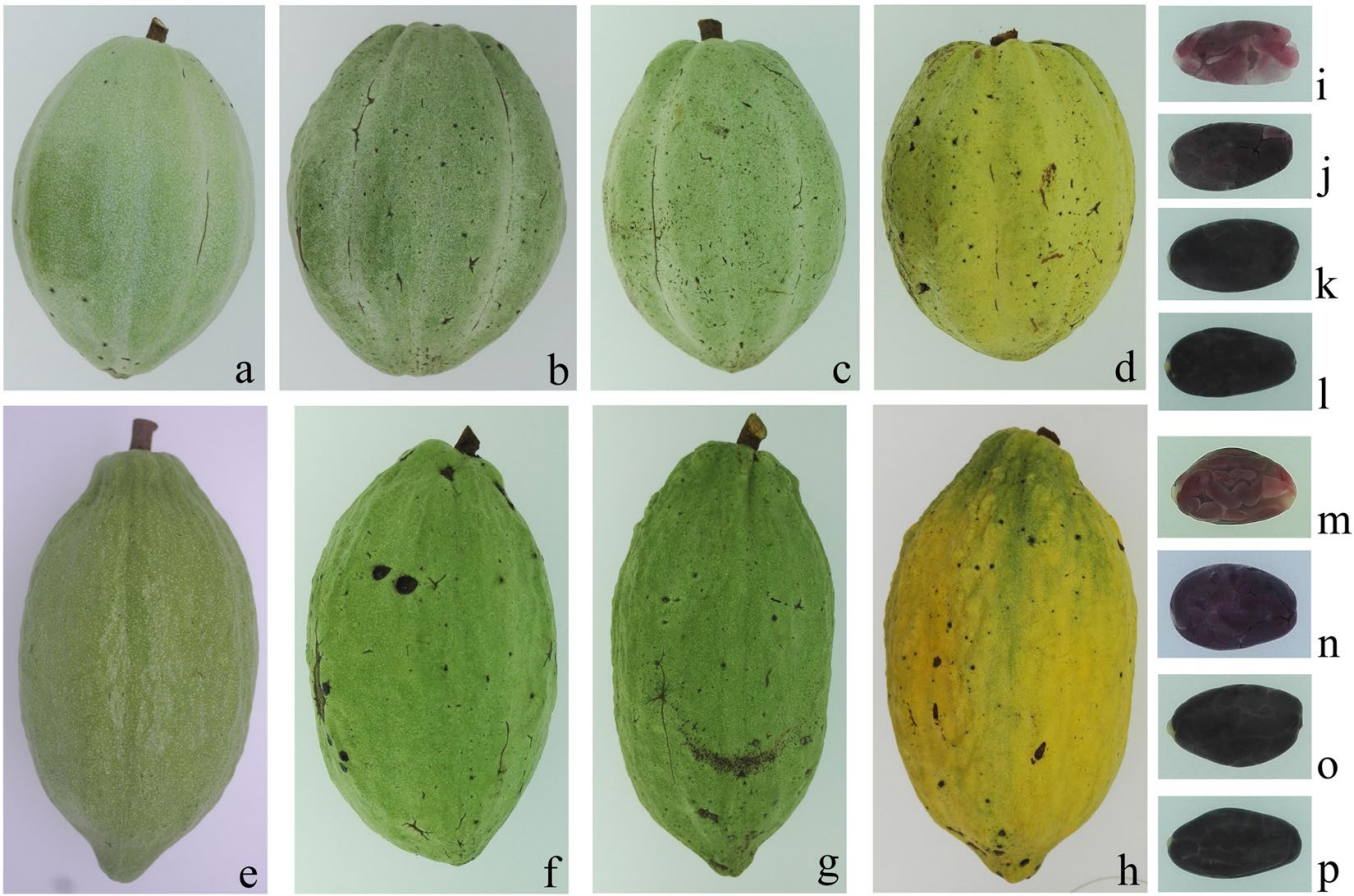
Pods of *Theobroma cacao* L. TAS42 and TAS57 used in transcriptional sequencing. (**a**–**d**) TAS42 pods at 105, 126, 147, and 168 DAP, respectively. (**e**–**h**) TAS57 pods at 105, 126, 147, and 168 DAP, respectively. (**i**–**l**) TAS42 seeds at 105, 126, 147, and 168 DAP, respectively. (**m**–**p**) TAS57 seeds at 105, 126, 147, and 168 DAP, respectively.

**Figure 2:**
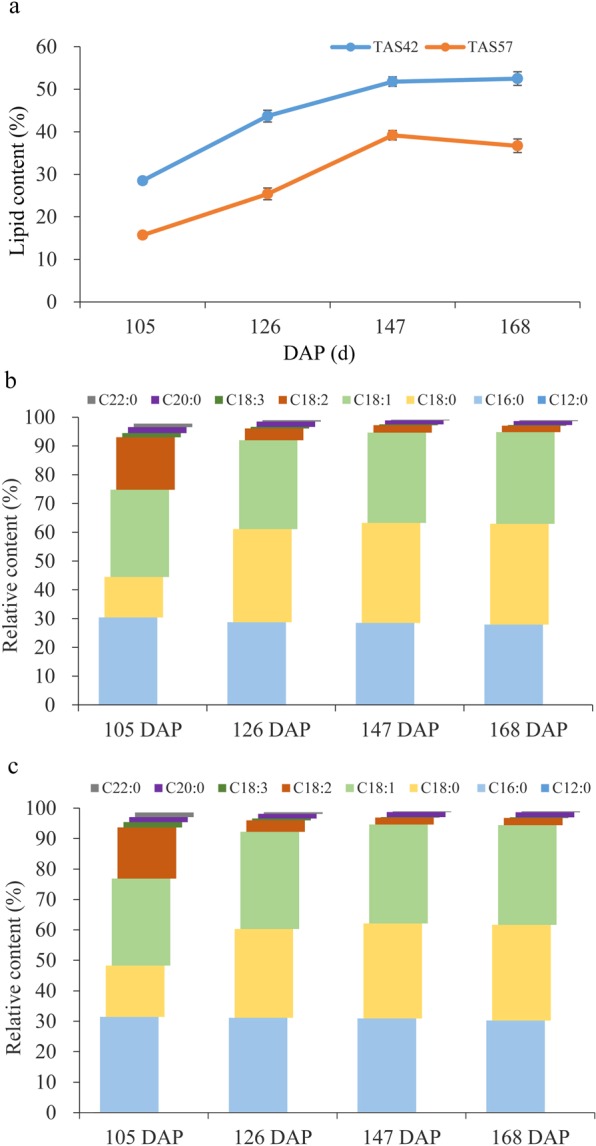
Changes in the lipid content and fatty acid composition of TAS42 and TAS57 during pod development. (**a**) Patterns of lipid accumulation in the developing seed. (**b**) Changes in fatty acid composition of the TAS42 seed during pod development. (**c**) Changes in fatty acid composition of the TAS57 seed during pod development. Values are the means of three biological replicates. C12:0, lauric acid; C16:0, palmitic acid; C18:0, stearic acid; C18:1, oleic acid; C18:2, linoleic acid; C18:3, alpha-linolenic acid; C20:0, arachidic acid; C22:0, behenic acid.

In this study, we first collected the seeds of 65 *T*. *cacao* accessions and analysed their lipid content, ranging from approximately 30–60% of dry weight (data not shown). Then, two different lipid content accessions, TAS42 and TAS57, were selected for further research. The pod and seed development processes in the high (TAS42) and low (TAS57) lipid content accessions were the same. The immature pods are green, and the colour of the seeds gradually turns from light purple to dark (Fig. 1a–p). Under field conditions, the pods of the two accessions completed their development and maturation in approximately 165 DAP. To assess the dynamic lipid accumulation patterns in developing seeds, we evaluated the TAS42 and TAS57 seed lipid contents at different developmental stages. Significant global and developmental stage-specific changes in fatty acid concentrations, saturation levels, and lipid accumulation were found (Fig. 1). The seed lipid was mainly composed of saturated fatty acids such as palmitic acid (C16:0) and stearic acid (C18:0) and unsaturated fatty acids such as oleic acid (C18:1) and was up to more than 90% after 126 DAP. Linoleic acid (C18:2) and alpha-linolenic acid (C18:3) decreased from 105 to 126 DAP and then stabilized along with pod maturation. However, other fatty acids such as palmitoleic acid (C16:1), arachidic acid (C20:0), and behenic acid (C22:0) stabilized at low content throughout pod development (Fig. 2).

### Digital gene expression (DGE) library sequencing and mapping

A total of sixteen DGE libraries were constructed to identify differences in gene expression levels. The sixteen libraries included four developmental stages of cacao cultivar TAS42 (designated as TAS42–105, 126, 147, 168) and four developmental stages of cacao cultivar TAS57 (designated as TAS57-105, 126, 147, 168), with two replicates each. Using Illumina sequencing, over 13 million raw reads were obtained in each library (Table 1). After reads containing adapters, reads containing poly-N sequences, and low-quality reads were removed, the total clean reads per library ranged from 10.1 to 12.8 million. The total reads mapped to the cacao cv. B97-61/B2 genome according to the HISAT2 analysis ranged from 8.6 to 12.3 million, and more than 73% of the reads had perfect matches and unique matches to reference genes (Table 1).

**Table 1:** Statistics of DGE sequencing of 16 libraries of different lipid content cacao accessions at various seed developmental stages.

	Raw reads	Clean reads (total reads)	Total mapped		Perfect match		Multiple match	
	Number	Number	Number	% of number	Number	% of number	Number	% of number
TAS42-105_1	13,503,598	10,299,473	9,014,098	87.52	8,203,192	79.65	810,881	7.87
TAS42-105_2	13,045,493	12,708,920	12,203,105	96.02	11,693,056	92.01	509,419	4.01
TAS42-126_1	15,198,848	11,484,049	9,896,953	86.18	8,986,849	78.26	910,434	7.93
TAS42-126_2	13,046,012	12,690,297	12,268,979	96.68	11,719,276	92.35	549,334	4.33
TAS42-147_1	14,886,461	11,468,870	9,797,856	85.43	8,701,046	75.87	1,097,114	9.57
TAS42-147_2	13,045,896	12,706,628	12,189,468	95.93	11,401,699	89.73	788,211	6.20
TAS42-168_1	16,770,028	12,765,857	11,011,828	86.26	9,845,749	77.13	1,166,446	9.14
TAS42-168_2	13,045,950	12,697,208	12,082,663	95.16	11,352,444	89.41	730,349	5.75
TAS57-105_1	13,045,861	12,701,364	12,167,907	95.80	11,592,681	91.27	575,805	4.53
TAS57-105_2	13,046,134	12,702,860	12,141,394	95.58	11,571,884	91.10	569,453	4.48
TAS57-126_1	17,966,147	11,752,498	10,150,633	86.37	8,716,950	74.17	1,434,022	12.20
TAS57-126_2	13,045,696	12,669,650	12,170,466	96.06	11,536,747	91.06	633,703	5.00
TAS57-147_1	17,248,764	11,148,327	9,781,542	87.74	8,409,930	75.44	1,371,817	12.31
TAS57-147_2	13,045,505	12,699,105	12,249,557	96.46	11,591,944	91.28	658,038	5.18
TAS57-168_1	15,385,137	10,070,435	8,637,412	85.77	7,400,806	73.49	1,236,221	12.28
TAS57-168_2	13,045,974	12,699,418	11,947,612	94.08	11,198,195	88.18	749,353	5.90

### Analysis of differential gene expression

**Figure 3:**
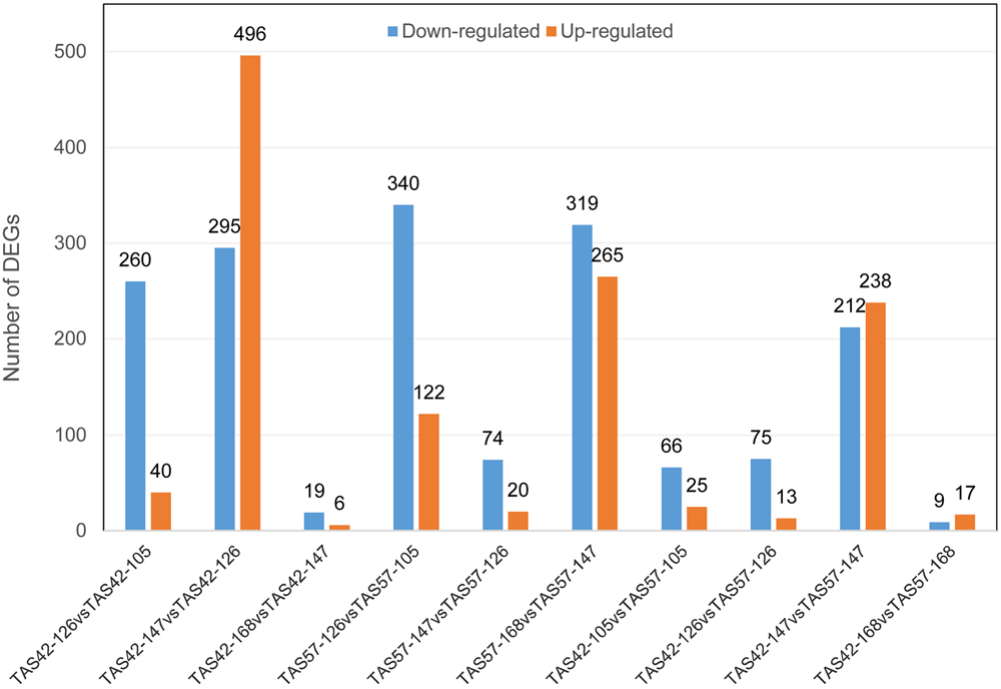
Transcripts differentially expressed between different seed developmental stages. Up- and downregulated transcripts were quantified. The results of ten comparisons between the two accessions are shown.

To compare the changes in gene expression between different accessions and different stages of maturation, fragments per kilobase of transcript per million mapped reads (FPKM) was introduced to calculate the expression level of each gene. All uniquely mapped reads were used to calculate the FPKM values of the genes. FPKM ≥1 or <1 was used as the threshold to identify whether a gene was expressed or not (Table 2). Biological replicates were necessary for high-throughput sequencing to obtain reliable analysis results. Our experiments showed a close relationship for each pair of biological replicates (Supplementary Fig. S1). The final FPKM of one gene with replicates in the same condition was the average value for all replicate data. The differentially expressed genes (DEGs) were hierarchically clustered based on the log_10_FPKM of the eight treatments, showing the overall gene expression pattern. The red bands indicate high gene expression, and the blue bands indicate low gene expression (Supplementary Fig. S2). The genes whose expression differed in the two samples were identified and filtered for fold-change ≥2.0 and diverge probability ≥0.8. The DEGs were compared between different stages within an accession and between accessions within a specific stage. The number of DEGs among these comparisons varied; approximately 25–791 genes displayed significant changes in expression, with an average of 291 DEGs. The numbers of upregulated and downregulated genes are shown in Fig. 3.

**Table 2:** Statistics for the gene numbers in the different FPKM intervals.

FPKM Interval	0–1	1–3	3–15	15–60	>60
TAS42-105_1	4092 (22.25%)	2874 (15.62%)	6522 (35.46%)	3610 (19.62%)	1297 (7.05%)
TAS42-105_2	6839 (31.25%)	2857 (13.06%)	6448 (29.47%)	4181 (19.11%)	1558 (7.12%)
TAS42-126_1	4553 (25.47%)	3145 (17.60%)	6517 (36.46%)	2771 (15.50%)	887 (4.96%)
TAS42-126_2	6980 (34.54%)	3149 (15.58%)	6465 (31.99)	2690 (13.31%)	924 (4.57%)
TAS42-147_1	4460 (25.29%)	2781 (15.77%)	5766 (32.70%)	3336 (18.92%)	1292 (7.33%)
TAS42-147_2	7938 (35.73%)	3013 (13.56%)	6051 (27.23%)	3737 (16.82%)	1480 (6.66%)
TAS42-168_1	4522 (24.96%)	2818 (15.55%)	5902 (32.58%)	3472 (19.16%)	1404 (7.75%)
TAS42-168_2	9449 (39.20%)	3197 (13.26%)	6155 (25.54%)	3837 (15.92%)	1465 (6.08%)
TAS57-105_1	6917 (30.71%)	2913 (12.93%)	6515 (28.92%)	4549 (20.20%)	1631 (7.24%)
TAS57-105_2	7188 (31.35%)	3010 (13.13%)	6545 (28.55%)	4554 (19.86%)	1629 (7.11%)
TAS57-126_1	4559 (24.63%)	3157 (17.06%)	6593 (35.62%)	3133 (16.93%)	1067 (5.76%)
TAS57-126_2	6848 (32.77%)	3481 (16.66%)	6569 (31.43%)	2938 (14.06%)	1062 (5.08%)
TAS57-147_1	4711 (26.91%)	3024 (17.27%)	6138 (35.06%)	2715 (15.51%)	918 (5.24%)
TAS57-147_2	6825 (32.72%)	2973 (14.25%)	6386 (30.62%)	3455 (16.57%)	1217 (5.84%)
TAS57-168_1	4098 (23.29%)	2736 (15.55%)	5742 (32.63%)	3613 (20.53%)	1407 (8.00%)
TAS57-168_2	7608 (35.37%)	2833 (13.17%)	5806 (26.99%)	3722 (17.30%)	1542 (7.17%)

**Figure 4:**
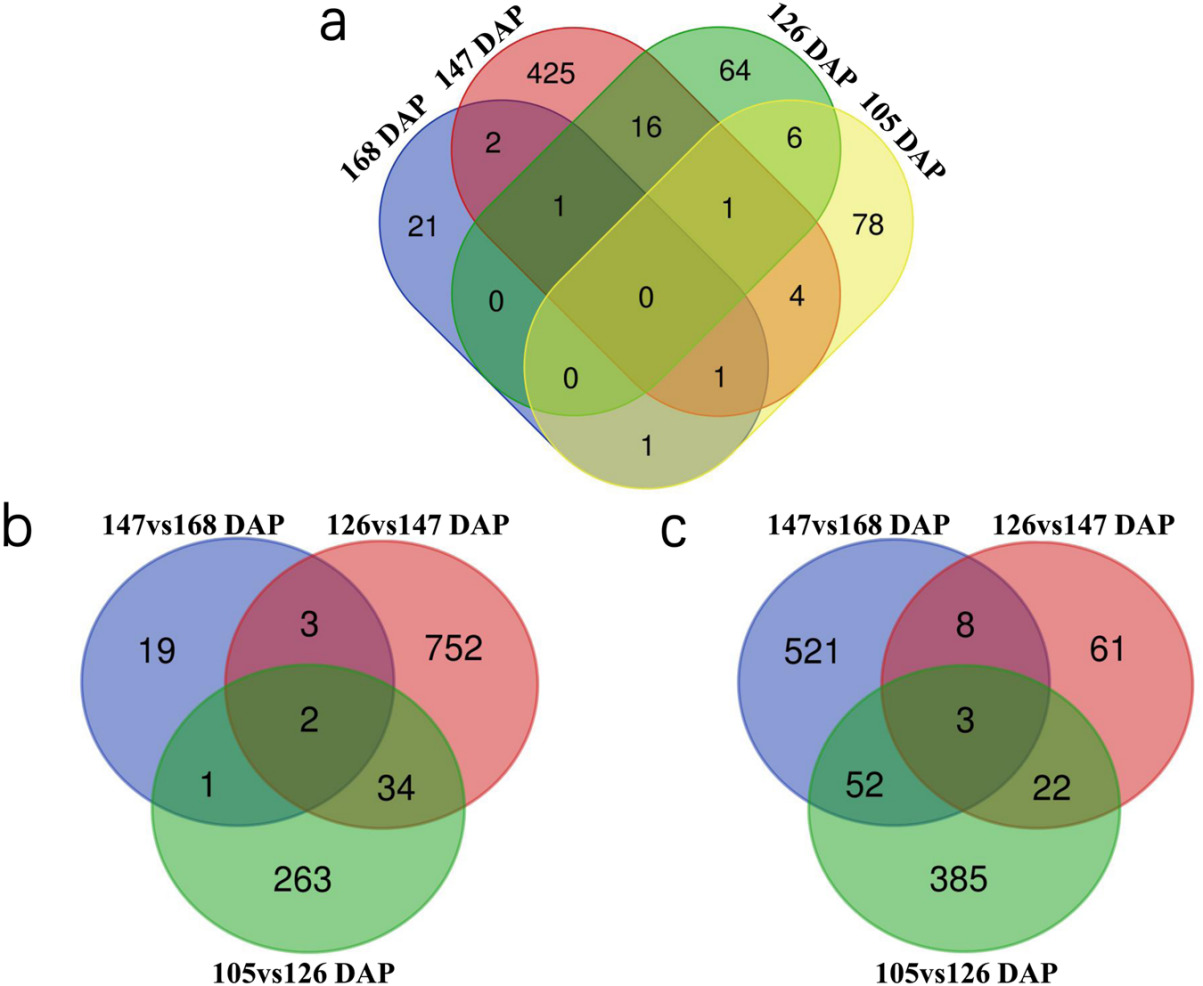
Venn diagram of DEGs in different seed developmental stages. (**a**) Distribution of DEGs in different seed developmental stages between accessions TAS42 and TAS57, (**b**) Distribution of DEGs in different seed developmental stages of accession TAS42, (**c**) Distribution of DEGs in different seed developmental stages of accession TAS57.

A Venn diagram was used to obtain the overlapping DEGs in the comparison group to see which DEGs were induced transiently and which DEGs were induced over a relatively long period of time. In this experiment, a comparative analysis of DEGs in the four developmental stages identified 78, 64, 425, and 21 DEGs at 105, 126, 147 and 168 DAP, respectively (Fig. 4a). Six genes were induced at both 105 and 126 DAP, 16 genes were induced at both 126 and 147 DAP, and 2 genes was induced at both 147 and 168 DAP, indicating that these genes may play important roles in seed development. (Fig. 4a). As shown in the Venn diagram, both TAS42 and TAS57 showed differential gene expression across seed development (Fig. 4b,c).

To illustrate the DEGs detected in the different developmental stages of pods from the high and low lipid content accessions, Gene Ontology (GO) enrichment analysis was performed to classify the gene functions. The DEGs were clustered into three main GO classification categories: Biological Process, Cellular Component and Molecular Function (Supplementary Fig. S3). The genes from the different expression clusters associated with the different functional categories clearly indicate the biological and molecular events involved in cacao seed maturation. In addition, ninety-five DEGs detected along seed development were associated with the cardinal enriched terms of fatty acid biosynthetic process, fatty acid metabolic process, lipid biosynthetic process, and carboxylic acid metabolic process (Table 3). Especially, genes differentially detected in TAS57 from 147 DAP to 168 DAP were largely enriched in the biological process domain, with the cardinal term metabolic process. In contrast, very few genes differentially detected in TAS42 from 147 DAP to 168 DAP were found to be enriched in a particular domain (Supplementary Table S1). Kyoto Encyclopedia of Genes and Genomes (KEGG) pathway enrichment analysis of the DEGs revealed the most representation effects on starch and sucrose, pyruvate, glycerolipid, glycerophospholipid, alpha-linolenic acid, arachidonic acid, amino acids, and secondary metabolite biosynthesis pathways. These annotations provide clues for investigating specific processes, especially those involved in starch and sucrose metabolism, fatty acid biosynthesis and glycerolipid metabolism.

**Table 3:** GO classification of fatty acid and lipid-related DEGs.

GO term	Gene ID	Gene number
Fatty acid biosynthetic process	18600369, 18600368	2
Fatty acid synthase activity	18588657, 18612736, 18609697	3
Fatty acid metabolic process	18601170, 18612736, 18589496, 18589082, 18588657, 18588068, 18607121, 18592535, 18600369, 18609697, 18600368, 18588970, 18606735	13
Carboxylic acid biosynthetic process	18598513, 18600369, 18592127, 18600368	4
Carboxylic acid metabolic process	18601193, 18601170, 18589496, 18611131, 18589082, 18597973, 18588852, 18606504, 18588657, 18591982, 18588068, 18609697, 18612736, 18590469, 18598513, 18592535, 18607121, 18600369, 18600368, 18607305, 18606735, 18592127	22
Cellular lipid metabolic process	18601170, 18612736, 18589496, 18611131, 18589082, 18588657, 18588068, 18607121, 18592535, 18600369, 18609697, 18600368, 18610613, 18611610, 18595095, 18588970, 18606735	17
Lipid biosynthetic process	18595095, 18600369, 18600368	3
Lipid metabolic process	18610613, 18611610, 18595095, 18612736, 18588068, 18589496, 18600369, 18600368, 18601170, 18611131, 18589082, 18588657, 18607121, 18592535, 18609697, 18588970, 18606735, 18614599	18
Lipid binding	18587333, 18611574, 18611493	3
Phospholipid binding	18587333, 18611574	2
Lipid localization	18588820, 18588654, 18588819, 18614349, 18602227, 18612639, 18597656, 18595117	8

### Fatty acid biosynthesis-related genes

Plant fatty acid biosynthesis occurs in plastids and is performed by a FAS dissociable complex of monofunctional enzymes. During cacao seed maturation, we found 34 DEGs involved in almost all parts of fatty acid biosynthesis and metabolism including *ACACA* (2 genes), *Fab* (6 genes), *FAT* (2 genes), *ADH* (2 genes), *OPR* (1 gene), *LOX* (6 genes), *EPHX* (5 genes), *PECR* (1 gene), *KCS* (1 gene), *ADH* (1 gene), methyltransferases and benzoate carboxyl methyltransferase (Table 4, Supplementary Tables S2, 3 and 4). Among the different seed developmental stages of TAS42, all of the 17 DEGs encoding fatty acid biosynthesis and metabolism showed significant changes in expression from 126 DAP to 147 DAP (Supplementary Table S3), while most of the 23 DEGs encoding fatty acid biosynthesis and metabolism in seeds of the low lipid content accession TAS57 showed significant changes in expression from 147 DAP to 168 DAP (Supplementary Table S4). There were almost 10 unique DEGs involving unsaturated fatty acid metabolism in TAS57, including *LOX*, *EPHX*, methyltransferases and benzoate carboxyl methyltransferase (Supplementary Table S4).

**Table 4:** Differentially expressed fatty acid synthesis-related genes between cacao accessions TAS42 and TAS57.

Gene_id	log_2_ Fold change TAS42/TAS57				Gene symbols	Annotation
	105 DAP	126 DAP	147 DAP	168 DAP		
18589496	0.4266	−0.3678	−3.7682	0.0760	ACACA	Biotin carboxylase 1
18612736	−0.0751	−0.4195	−3.4359	0.2181	FATA	FatA acyl-ACP thioesterase
18586907	−0.9839	0.1711	−4.0663	0.1983	PECR	3-oxo-5-alpha-steroid 4-dehydrogenase
18603880	3.2412	1.1221	1.1539	0.8392	ALDH	Aldehyde dehydrogenase 2B4
18600369	0.1827	0.5471	6.9570	−0.4001	LOX1_5	Linoleate 9S-lipoxygenase 6
18600366	−1.7522	0.3061	7.6223	−0.6442	LOX1_5	Lipoxygenase family protein
18600368	−1.0429	0.5187	7.5498	−0.5974	LOX1_5	Linoleate 9S-lipoxygenase 5
18600000	−4.9947	−1.6724	1.5236	−1.6439		Uncharacterized protein
18589945	1.8140	0.7842	−3.3797	1.2651	BAMT	Benzoate carboxyl methyltransferase
18589726	0.5753	1.5502	−3.5833	1.3138	LOX2S	Linoleate 13S-lipoxygenase 2
18610880	1.8506	0.0880	−8.4236	−0.9556	LOX2S	Salicylate O-methyltransferase
18585926	−0.8215	2.2386	−3.4894	−0.9676		Methyltransferases superfamily protein
18610882	−2.2543	−1.9473	−8.1930	−2.5850	BAMT	Benzoate carboxyl Methyltransferase
18585925	2.7960	−4.1084	−5.1181	−5.1606		Methyltransferases superfamily protein
18585923	1.5566	0.2636	−1.8789	−4.9523		Uncharacterized protein
18608686	−1.4438	−2.6428	−2.6792	2.3925	EPHX2	Hydrolases superfamily protein

### TAG assembly-related genes

TAG biosynthesis occurs in the endoplasmic reticulum through an acyl-CoA-dependent pathway and acyl-CoA-independent pathway. In the acyl-CoA-dependent pathway, acyl-CoA is used as a substrate for the serial incorporation of three acyl groups into the glycerol backbone, dependent on enzymes such as glycerol-3-phosphate acyltransferase (GPAT), lysophosphatidic acid acyl transferase (LPAT), phosphatidic acid phosphatase (PAP), and diacylglycerol acyltransferase (DGAT). In the acyl-CoA-independent pathway, phospholipids are used as acyl donors and diacylglycerol (DAG) as an acceptor, and phospholipid/diacylglycerol acyltransferase (PDAT) is used for TAG production. In the present study, we found differential expression of 1 gene coding for *GPAT*, 2 for *LPAT* and 6 for *PDAT* in the seed, suggesting that the acyl-CoA-independent pathway may be the most important pathway for the synthesis of TAGs in *T*. *cacao*. Five *PDAT* genes were detected in both accessions examined, among which 2 genes (18609038 and 18598114) showed a significant increase in expression from 105 DAP to 126 DAP and 2 genes (18608303, 18611047) showed a significant increase in expression from 147 DAP to 168 DAP (Supplementary Tables S5, 6 and 7). Moreover, the expression level of *late embryogenesis abundant* (*LEA*), *zinc finger protein* (*ZNF3*) and methyltransferase genes significantly changed over the course of seed development, and these genes have been shown to participate in lipid body formation and lipid synthesis regulation^16^. We found 7 DEGs involved in glycerolipid and glycerophospholipid metabolism in the comparison between TAS42 and TAS57. The expression level of *aldehyde dehydrogenase* (*ALDH*) in TAS42 was higher than that in TAS57 along seed development. The expression levels of *PDAT* (18611047) and *glycerol-3-phosphate dehydrogenase* (*GPDH*) in TAS42 were lower than those in TAS57 at stage 105 DAP to 126 DAP. However, in stages 147 DAP and 168 DAP, the expression levels of *PDAT* (18611047) and *GPDH* in TAS42 were higher than those in TAS57 (Table 5, Supplementary Table S5).

**Table 5:** Differentially expressed TAG synthesis-related genes between cacao accessions TAS42 and TAS57.

Gene_id	log_2_ Fold change TAS42/TAS57				Gene symbols	Annotation
	105 DAP	126 DAP	147 DAP	168 DAP		
18603880	3.2412	1.1221	1.1539	0.8392	ALDH	Aldehyde dehydrogenase
18588216	−4.1924	−1.3686	−12.3719	0.9386	PDAT	phospholipid:diacylglycerol acyltransferase
18597445	1.8600	0.8677	−3.6121	−0.1165	AKR1B	NAD(P)-linked oxidoreductase
18611047	−2.9069	−1.9511	6.6342	−0.0180	PDAT	GDSL esterase/lipase
18589337	−1.1971	0.2729	−6.2253	−1.4686	ZNF3	Zinc finger protein 3
18604975	−3.6267	−2.5474	−1.8126	−1.2393	ADPRM	ADP-ribose/CDP-alcohol diphosphatase
18600884	−0.0848	0.1355	−5.6107	0.9720	GPDH	Glycerol-3-phosphate dehydrogenase (NAD+)

### Quantitative RT-PCR (qRT-PCR) analysis of genes

**Figure 5:**
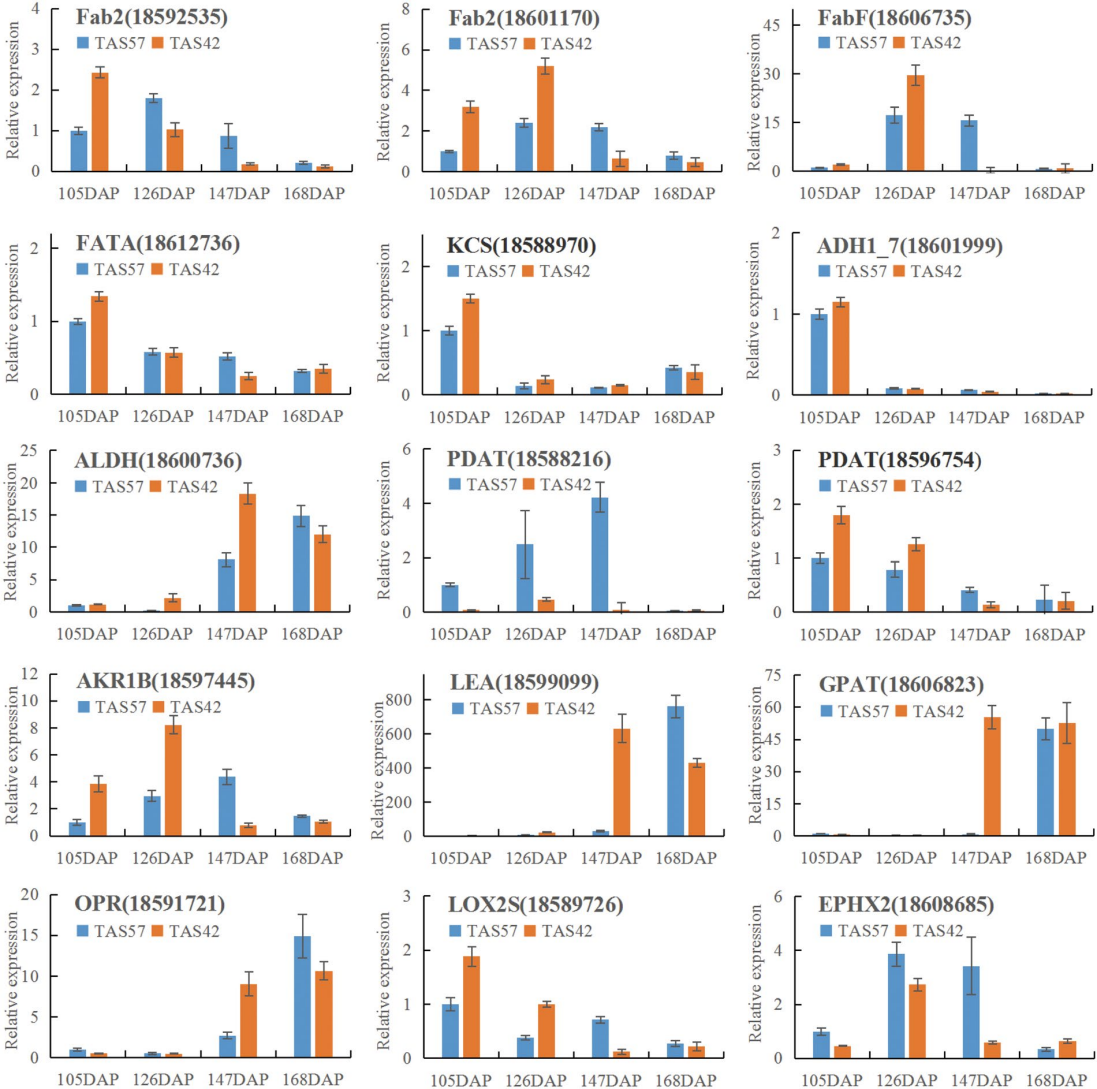
Expression analysis of 15 candidate DEGs related to lipid biosynthesis metabolism in the cacao seed by qRT-PCR. The Y-axis represents the relative expression, and the X-axis depicts the pod maturation stages. The experiment was repeated three times, and the resulting data are presented with error bars, with *n* = 3.

To confirm the reliability of the RNA sequencing (RNA-Seq) results and further analyse the differences in the expression profiles, fifteen genes related to fatty and lipid metabolism were chosen for qRT-PCR analysis, including 18592535 (Fab2), 18601170 (Fab2), 18606735 (FabF), 18612736 (FATA), 18588970 (KCS), 18601999 (ADH1_7), 18600736 (ALDH), 18588216 (PDAT), 18596754 (PDAT), 18597445 (AKR1B), 18599099 (LEA), 18606823 (GPAT), 18591721 (OPR), 18589726 (LOX2S), and 18608685 (EPHX2). The results showed that, although there were fold changes in gene expression among different seed developmental stages, the expression patterns determined for all fifteen genes were consistent, confirming the trends observed in the RNA-Seq results (Fig. 5).

## Discussion

The cacao, a worldwide cash crop for chocolate and cosmetic industries, has a high quality and unique physicochemical characteristics. The genome sequences of cacao have been completed, and high-throughput RNA-Seq is an effective method to obtain large amounts of transcriptome data and DEGs from different types. In this work, the kinetic patterns of lipid contents and fatty acid compositions were detected at different developmental stages of cacao pods, and the optimal accessions for comparative deep transcriptomic analysis were determined. The transcriptomes of different lipid content cacao seeds were sequenced by Illumina technology, and the DEGs were functionally annotated. The DEGs involved in lipid accumulation of developing pods were determined by the NOISeq method, and the role and regulation of some key genes was analysed.

Lipid accumulates in cacao seeds at a slow rate in the first 4 months of pod development and then accelerates into a rapid, linear rate after the seeds solidify until near the beginning of maturation, when lipid build-up proceeds at a greatly reduced rate^8^. The most noticeable changes concerned the percentage of linoleic acid (C18:2) between 105 and 126 DAP, which dropped from 16% to 3%. After 126 DAP, the amount of lipid in the seeds continued to increase. The fatty acid composition did not exhibit a significant change, and, importantly, palmitic acid (C16:0), stearic acid (C18:0) and oleic acid (C18:1) were the main components, the relative ratio of which was maintained during the examined developmental period (Fig. 2). These trends in fatty acid storage and accumulation are very different from those observed in other tropical wood lipid plants^15,16^. For example, in coconut, medium-chain fatty acids (C8-C14) predominate the lipid components^16^. These differences indicate that cacao seeds possess species-specific mechanisms for lipid accumulation.

In the last decade, the details of fatty acid and lipid biosynthesis derived from plant seeds have generally suggested that the substrate specificity of FatA and FatB acyl-ACP thioesterases, to a large extent, determine the fatty acid composition of storage lipids^17,18^. FatA is generally accepted to act on long-chain acyl-ACPs, preferentially on oleoyl-ACP, while FatB preferably hydrolyses acyl-ACPs with saturated fatty acyl chains^19,20^. In the present study, one *FatA* and one *FatB* gene were identified in the transcriptomes of the developing cacao seed. Gene duplication and neofunctionalization are common features among species that play a key role in driving evolutionary novelty for organisms to differentiate new organs or synthesize various storage substances^21,22^. *FatB* genes mainly act to form medium-chain fatty acids of lauric acid (C12:0) and myristic acid (C14:0) in oil palm and coconut^15,16^. As in tropical crops, the *FatB* isoform leading to long-chain fatty acid accumulation of palmitic acid (C16:0) and stearic acid (C18:0) was expressed in cacao seeds. These findings suggest that, in cacao, to lengthen the acyl chain, the cleavage of acyl-ACP requires the fatty acid-specific *FatB* gene. Previous investigations have indicated that synthesis of fatty acids and supply of pyruvate rather than acyl assembly into TAG might be the major factors that control lipid storage^23–25^. Our transcriptomic data found DEGs involved in almost all parts of fatty acid biosynthesis and metabolism including *ACACA*, *Fab*, *FAT*, *ADH*, *OPR*, *LOX*, *EPHX*, *PECR*, *KCS*, and *ADH* (Table 3). However, the expression patterns of these DEGs were significantly different; the sharp change in the expression level in TAS57 was delayed from that observed in TAS42. The *WRINKLED1* (*WRI1*) transcription factor acts as a coregulator of genes involved in fatty acid biosynthesis, especially at early seed stages, and *WRI1* expression is vital in the carbon flux necessary for the synthesis of fatty acids in seeds^26–29^. We also found that *WRI1* showed the delayed sharp change in the expression level in TAS57 than TAS42. (Supplementary Table S6, 7). During the last seed maturation stage, the lipid content of TAS57 decreased from 39.2% to 36.7%. Notably, lipid degradation plays an indispensable role in whole plant growth and the development process^30–32^. These results, combined with the results from other crops, suggest that fatty acid biosynthesis hysteresis and lipid degradation are the most important factors leading to lipid content reduction.

Generally, fatty acid synthesis may be accomplished by releasing the fatty acid from the acyl-ACP molecule by acyl-ACP thioesterases (FATA and FATB). The unsaturated bonds on the monounsaturated fatty acids at specifically defined positions are catalysed by SAD to form unsaturated fatty acids^24^. Interestingly, oleic acid (C18:1) and linoleic acid (C18:2) are the main unsaturated fatty acids of cacao seeds^10,33^. The cacao genome has been shown to contain eight *SAD* genes^1,4^. Among the eight *SAD* genes, *TcSAD1* and *TcSAD7* are the primary ones expressed in developing seeds, with *TcSAD7* being more highly expressed over all stages except the maturation stage, implying that the activities of both TcSAD1 and TcSAD7 are involved in the synthesis and accumulation of C18:1 in cacao^4^. In our study, *TcSAD7* (18592535) was detected in both TAS42 and TAS57 and exhibited a downward expression trend along seed development. At the onset of lipid accumulation, the total percentage of mainly unsaturated fatty acids (C18:1 + C18:2 + C18:3) was highest. The major fatty acid composition shift occurred between 105 and 126 DAP when the transiently high proportion of C18:2 dropped rapidly to approximately 3%. C18:2 synthesis catalysed by C18:1 desaturase ceased during this period, as indicated by the increase in dry seed weight with a decrease in C18:2 content^10^. Although the biochemical mechanism has not been clarified, C18:2 content may limit the activity potential of TcSAD and activate the termination of C18:2 synthesis, and, during the following stages, the ratio of unsaturated fatty acids and saturated fatty acids was maintained. The ratio of mainly fatty acids should be investigated in future studies.

## Conclusions

Illumina sequencing was performed on messenger RNAs (mRNAs) obtained from developing cacao seeds. The comparison of high and low lipid content cacao accessions by RNA-Seq and qRT-PCR of genes involved in fatty acid synthesis, TAG accumulation and metabolism led to the hypothesis that fatty acid biosynthesis hysteresis and lipid degradation are the primary factors leading to lipid content reduction. An isoform of the *Fab2* gene *TcSAD7* (18592535) was expressed at high levels at the onset of massive lipid accumulation in the seed, which may be responsible for the stable ratio of unsaturated fatty acids. TAG assembly enzymes (PDAT and GPDH) may result in the considerable differences in lipid content between TAS42 and TAS57. Future research will aim to verify the function of key candidate genes using transgenic approaches in model organisms. Furthermore, future studies must confirm the differentiated lipid hypothesis, for example, by assessing the metabolomics of the seed organ at different time points. The results may help to understand and manipulate the lipid contents of cacao by means of genetic engineering.

## Materials and Methods

### Plant material

The high lipid content cacao accession *T*. *cacao* (TAS42) and low lipid content cacao accession *T*. *cacao* (TAS57) were used in this study. The plants were grown in the Spice and Beverage Research Institute of Chinese Academy of Tropical Agricultural Science, Wanning, Hainan, China. According to the data from our laboratory, the pod development periods of the two cultivars are similar. During flowering period, self-bred was carried out for each cultivar. Seed samples were collected at four different developmental stages from self-bred pods: 105 days after pollination (DAP), 126 DAP, 147 DAP and 168 DAP. After collection, samples were flash-frozen in liquid nitrogen and stored at −80 °C before use. Owing to pod setting rate, each cacao cultivar successfully produced several self-bred pods, only satisfied two replicates of each seed developmental stages. In the following investigation, two replicates were used in the DEG analysis.

### Lipid content and fatty acid composition determination

The total lipid content in the cacao accessions was assayed according to the method by Bucheli *et al*. with minor modification^10^. Briefly, freeze-dried cacao seed powder (2–5 g) was poured into a round-bottom flask, followed by the addition of 60 mL chloroform-methanol (1:1, v/v) and homogenization for 1.5–2.0 min. Successively, the mixture was reflux-heated in a water bath at 60 °C for 1 h and then cooled to room temperature. After being filtered through a fluted filter paper, the resultant filtrate was collected and dried under reduced pressure using a vacuum evaporator. Then, the lipid obtained was resolved in 25 mL petroleum ether and mixed with 5 g sodium sulfate anhydrous to further remove the residual solvent. Subsequently, the mixture was separated by centrifugation at 3000 g for 5 min, and the supernatant was collected, evaporated and dried at 105 °C in succession. The residue was then weighed and stored at −20 °C. Approximately 10 mg of extracted lipid sample was dissolved in 2 mL of N-hexane with 2.5 mg/mL of methyl nonadecanoate in a tube, and 0.1 mL of methanolic potassium hydroxide solution (2 mol/L) was added. The tube was capped tightly and shaken vigorously for approximately 2 min. The solution was then centrifuged at 3000 g for 5 min, and 1 mL of N-hexane upper phase was transferred into a 2 mL autosampler vial, and 5 μL was injected onto a Chrompack CP-Sil88 column (100 m × 0.25 mm i.d., 0.25 μm run on a film thickness; Varian Inc., USA) for subsequent gas chromatography analysis.

### RNA isolation and quantification

A total of 16 samples (two replicates of two differential lipid content accessions at four time points) were prepared for RNA extraction. Total RNA was extracted individually using TRIzol reagent (Life Technologies). High-quality RNA was obtained through twice chloroform/isoamyl alcohol (24:1) purification and then mixed with approximately the same quantity. RNA purity was checked using a NanoPhotometer^®^ spectrophotometer (IMPLEN, CA, USA).

### Digital gene expression (DGE) library preparation and sequencing

A total of 5 μg of RNA per sample was used as the input material for the RNA sample preparations. Sequencing libraries were generated using the NEBNext^®^ Ultra^TM^ RNA Library Prep Kit for Illumina^®^ (NEB,USA) following the manufacturer’s recommendations. In brief, mRNA was purified from the total RNA using oligo (dT) magnetic beads. After fragmentation with divalent cations in NEBNext First Strand Synthesis Reaction Buffer (5×), first-strand cDNA was synthesized using random hexamer primers and M-MuLV Reverse Transcriptase (RNaseH^−^). Then, second-strand cDNA synthesis was performed using DNA polymerase I and RNaseH. To select cDNA fragments 150–200 bp in length, the library fragments were purified using the AMPure XP system (Beckman Coulter, Beverly, USA). PCR was then performed with Phusion High-Fidelity DNA polymerase, Universal PCR primers and an Index (X) Primer. Finally, the PCR products were purified (AMPure XP system), and the library quality was assessed on an Agilent Bioanalyzer 2100 system and ABI StepOnePlus Real-Time PCR System. The library was sequenced on the Illumina HiSeq. 2000 platform, and 100-bp paired-end reads were generated.

### Bioinformatics analysis

The sequence data sets are deposited in the NCBI Short Read Archive (SRA, http://www.ncbi.nlm.nih.gov/sra) under the accession number SRP136974 (SRR6928365-SRR6928380). Raw data had adaptor sequences and a few low-quality sequences, along with several types of impurities. Clean data were obtained by removing low-quality reads containing adapters or poly-N sequences from the raw data. All downstream analyses were based on clean, high-quality data.

The reference cacao genome (B97–61/B2) and gene model annotation files were downloaded from the genome website directly. All clean reads were aligned to the reference genome using the HISAT2-2.0.4 aligner^34^. To estimate the expression levels of each gene for the four pod developmental stages, the read numbers mapped to each gene were counted using HTSeq v0.5.4p3. And then normalized expression values of genes were estimated as the fragments per kilobase of transcript per million mapped reads (FPKM)^35^ by RSEM (v1.2.9)^36^. The differentially expressed genes (DEGs) were required to achieve the probability (P) thresholds of differential expression of 0.8 and a minimum two-fold change in expression between samples, as determined by NOISeq.^37^. A higher probability resulted in a remarkable change in expression between the two samples.

Gene Ontology (GO) functional enrichment analysis was implemented in the GOseq R package^38^, and Kyoto Encyclopedia of Genes and Genomes (KEGG) pathway enrichment analysis was performed to examine the high-level functions and utilities of the DEGs^39^. In all tests, P values were calculated using Benjamini-corrected modified Fisher’s exact test, and ≤0.05 was considered the threshold of significance.

### qRT-PCR validation

To verify the differential expression detected by Illumina RNA-Seq, qRT-PCR was performed on the same samples that had been used previously. A set of fifteen genes was chosen at random, the expression of each of which was checked at different time points and compared with their observed FPKM. qRT-PCR was performed by using an ABI QuantStudio™ 6 Flex Real-Time thermocycler (Applied Biosystems, Foster City, CA, USA) with the SYBR Green Real-Time PCR Master Mix (TOYOBO, Osaka, Japan) following the manufacturer’s instructions. The forward and reverse primers used for qRT-PCR are listed in Supplementary Table S8. The thermal cycling conditions were as follows: 2 min at 95 °C, followed by 45 cycles of denaturation at 95 °C for 10 s, annealing at 60 °C for 15 s, and extension at 72 °C for 30 s. Optical data were acquired following the extension step, and the PCRs were subject to melting curve analysis beginning at 60 °C through 95 °C at 0.1 °C s^−1^. Actin was chosen as the reference gene based on the study by Pinheirob *et al*.^40^. The data are presented as an average ± SD of three independently produced RT preparations used for PCR runs, each having at least three replicates. The relative expression levels were calculated using the delta-delta Ct method^41^.

## Supplementary information

Supplementary information
