# Supplementary material for: Transcriptional profiling reveals differentially expressed genes involved in lipid biosynthesis during cacao seed development
Source: Sci Rep. 2019 Nov 21;9:17263. doi: 10.1038/s41598-019-53959-9 (PMC6872657; doi:10.1038/s41598-019-53959-9)
Supplement: Supplementary file 1 — Supplementary information [file 41598_2019_53959_MOESM1_ESM.pdf]

Transcriptional profiling reveals differentially expressed genes involved in lipid biosynthesis during cacao seed development

Fupeng Li, Baoduo Wu, Lin Yan, Chaoyun Hao, Xiaowei Qin, Jianxiong Lai & Yinghui Song*

Spice and Beverage Research Institute, Chinese Academy of Tropical Agricultural Sciences/Key Laboratory of Genetic Resources Utilization of Spice and Beverage Crops, Ministry of Agriculture, Wanning 571533, P. R. China

* Corresponding author: Yinghui Song

Tel.: +86-898-62553687

Fax: +86-898-62561083

E-mail: labcacao@163.com

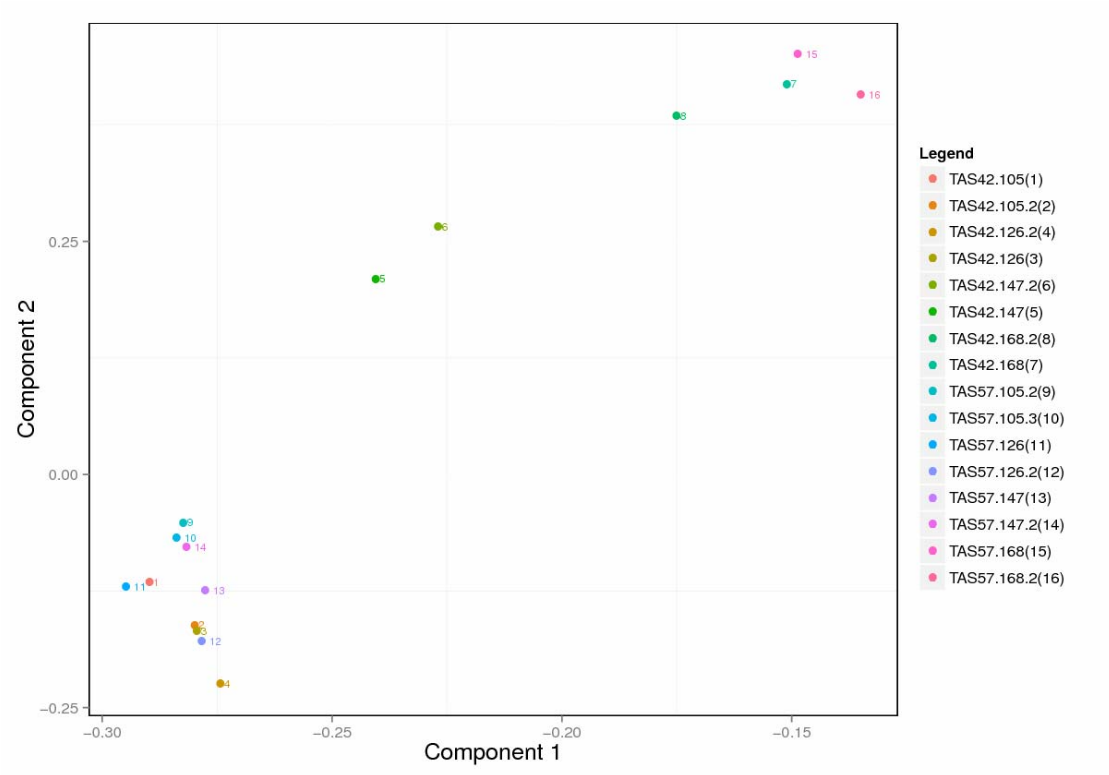

Figure S1 Visualization of the first two principal components separating samples according to their developmental stage.

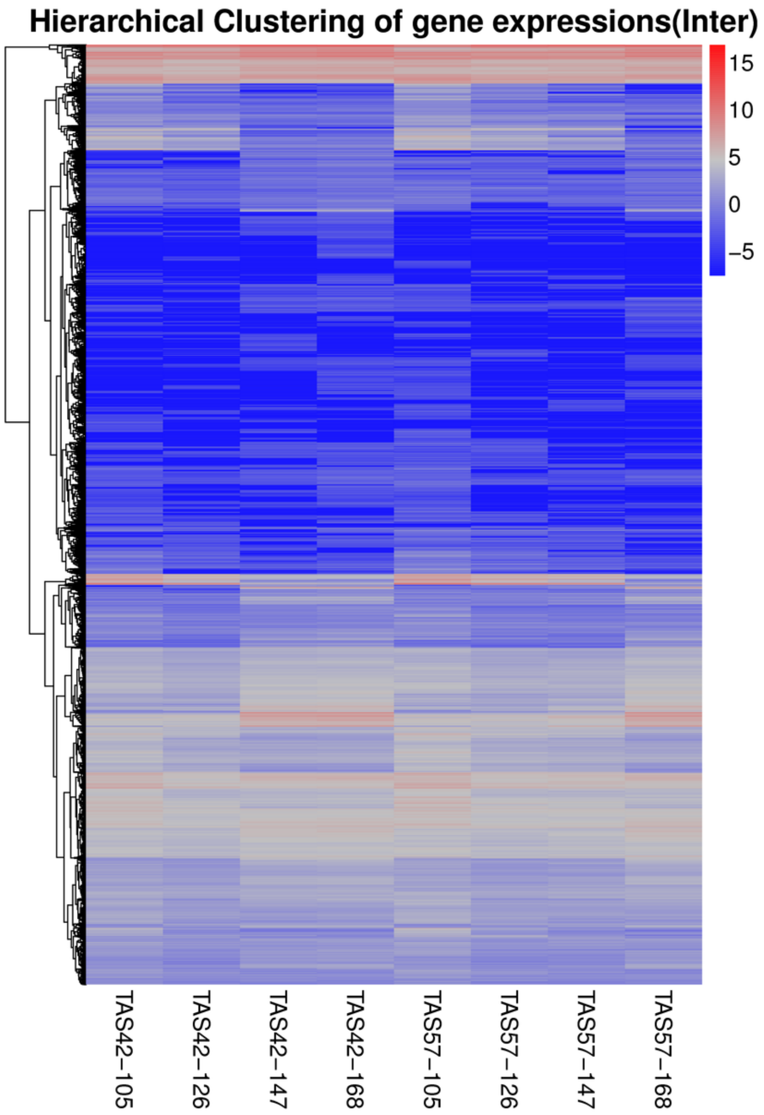

Figure S2 The expression level heatmap of all DEGs detected between TAS42 and TAS57 cacao seeds.

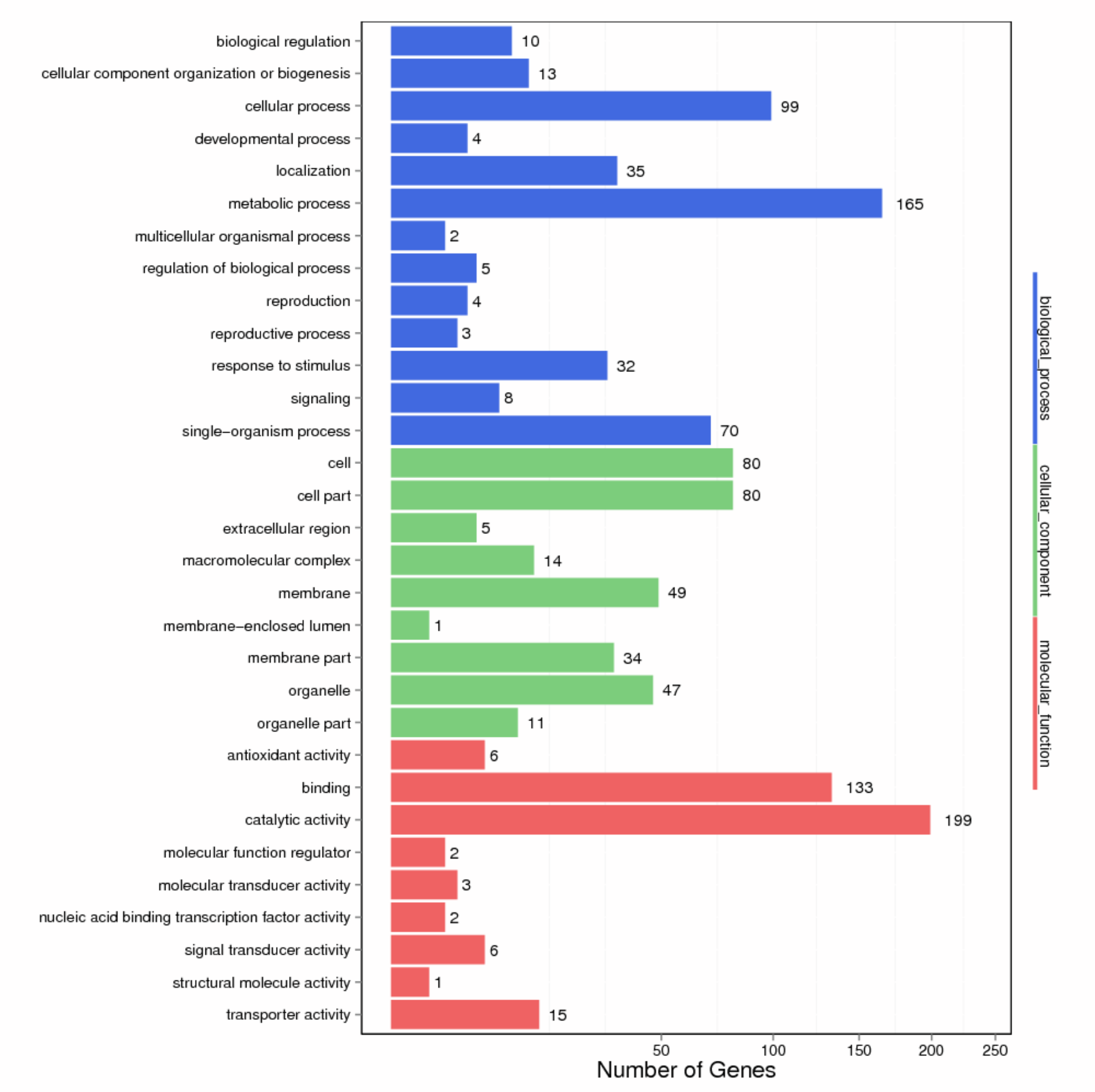

Figure S3 Histogram of GO classifications.

Table S1 GO classification and DEG statistics.

Gene Ontology		TAS42-105 vsTAS42-126	TAS42-126 vsTAS42-147	TAS42-147 vsTAS42-168	TAS57-105 vsTAS57-126	TAS57-126 vsTAS57-147	TAS57-147 vsTAS57-168	TAS57-105 vsTAS42-105	TAS57-126 vsTAS42-126	TAS57-147 vsTAS42-147	TAS57-168 vsTAS42-168
Bological process	biological phase	2	-	-	1	-	12	-	1	6	-
	biological regulation	4	10	1	6	-	-	2	-	-	-
	cellular component organization or biogenesis	19	13	-	11	4	7	-	1	5	-
	cellular process	50	99	1	70	11	61	9	9	44	1
	developmental process	1	4	-	1	-	2	1	-	2	-
	localization	14	35	-	20	5	25	2	4	17	-
	metabolic process	56	165	2	95	22	118	22	17	98	4
	multicellular organismal process	-	2	-	-	-	1	-	-	1	-
	multi-organism process	-	-	-	-	-	-	-	1	-	-
	negative regulation of biological process	2	-	-	2	-	3	-	1	1	-
	regulation of biological process	3	5	-	4	-	6	1	1	2	-
	reproduction	1	4	-	-	-	3	-	1	3	-
	reproductive process	-	3	-	-	-	2	-	1	2	-
	response to stimulus	3	32	-	7	1	20	5	2	14	2
	signaling	1	8	-	3	-	3	-	-	2	-
	single-organism process	24	70	1	33	7	44	2	6	33	1

Cellular component	cell	39	80	-	54	9	57	13	8	38	1
	cell part	39	80	-	54	9	57	13	8	38	1
	extracellular region	2	5	-	4	2	3	5	1	3	-
	macromolecular complex	14	14	-	10	3	8	3	2	5	-
	membrane	19	49	-	27	8	28	4	5	21	-
	membrane-enclosed lumen	1	1	-	-	-	-	-	-	-	-
	membrane part	11	34	-	18	4	19	4	3	14	-
	organelle	28	47	-	34	5	29	5	5	19	1
	organelle part	8	11	-	7	1	6	-	1	2	-
Molecular function	antioxidant activity	1	6	-	1	1	4	1	-	3	-
	binding	54	133	1	71	15	86	11	12	65	3
	catalytic activity	62	199	2	104	23	136	20	14	105	6
	molecular function regulator	2	2	-	4	-	5	-	1	2	-
	molecular transducer activity	1	3	-	-	-	2	-	-	1	-
	nucleic acid binding transcription factor activity	-	2	-	2	1	1	1	1	1	-
	signal transducer activity	1	6	-	5	-	1	-	-	2	-
	structural molecule activity	1	1	-	1	-	-	-	-	-	-
	transporter activity	3	15	-	4	1	9	1	2	7	-

Table S2 Differentially expressed fatty acid synthesis-related genes between cacao accessions TAS42 and TAS57.

Gene ID	TAS57 105 DAP	TAS42 105 DAP	log_2 Fold Change	P-value	Regulated	Name	Gene symbols	Annotation
18603880	7.8	73.8	3.24	0.83	Up	TCM_012037	ALDH	Aldehyde dehydrogenase 2B4
18600000	5.4	0.2	-4.99	0.81	Down	TCM_026098		Uncharacterized protein
Gene ID	TAS57 126 DAP	TAS42 126 DAP	log_2 Fold Change	P-value	Regulated	Name	Gene symbols	Annotation
18608686	323.9	51.9	-2.64	0.80	Down	TCM_008073	EPHX2	Hydrolases superfamily protein
18585925	13.2	0.8	-4.11	0.83	Down	TCM_042580		Methyltransferases superfamily protein
Gene ID	TAS57 147 DAP	TAS42 147 DAP	log_2 Fold Change	P-value	Regulated	Name	Gene symbols	Annotation
18589496	27.7	2.0	-3.77	0.81	Down	TCM_039756	ACACA	Biotin carboxylase 1
18612736	257.7	23.8	-3.44	0.82	Down	TCM_002853	FATA	FatA acyl-ACP thioesterase
18586907	27.8	1.7	-4.07	0.83	Down	TCM_044452	PECR	3-oxo-5-alpha-steroid 4-dehydrogenase
18589945	719.0	6.9	-3.38	0.80	Down	TCM_040854	BAMT	Benzoate carboxyl methyltransferase
18589726	116.7	9.7	-3.58	0.83	Down	TCM_040462	LOX2S	Linoleate 13S-lipoxygenase 2
18610880	257.5	0.8	-8.42	0.99	Down	TCM_000326	LOX2S	Salicylate O-methyltransferase
18585926	131.3	11.7	-3.49	0.82	Down	TCM_042582		Methyltransferases superfamily protein
18610882	16.1	0.1	-8.19	0.94	Down	TCM_000328	BAMT	Benzoate carboxyl methyltransferase
18600369	3.1	380.8	6.96	0.99	Up	TCM_026544	LOX1_5	Linoleate 9S-lipoxygenase 6
18600366	6.3	1250.2	7.62	0.99	Up	TCM_026539	LOX1_5	Lipoxygenase family protein
18600368	80.1	15007.2	7.55	0.99	Up	TCM_026543	LOX1_5	Linoleate 9S-lipoxygenase 5
18585925	10.9	0.3	-5.12	0.85	Down	TCM_042580		Methyltransferases superfamily protein
Gene ID	TAS57 168 DAP	TAS42 168 DAP	log_2 Fold Change	P-value	Regulated	Name	Gene symbols	Annotation
18585925	20.7	0.6	-5.16	0.88	Down	TCM_042580		Methyltransferases superfamily protein

Table S3 Differentially expressed fatty acid synthesis-related genes of cacao accession TAS42.

Gene ID	105 DAP	126 DAP	log_2 Fold Change	P-value	Regulated	Name	Gene symbols	Annotation
18595640	5.7	0.01	-9.15	0.86	Down	TCM_027898		DNA/RNA polymerases superfamily protein

Gene ID	126 DAP	147 DAP	log_2 Fold Change	P-value	Regulated	Name	Gene symbols	Annotation
18599284	74.7	8.3	-3.17	0.81	Down	TCM_025140	ACACA	Biotin carboxyl carrier protein subunit of Het-ACCase (BCCP1)
18589496	38.7	2.0	-4.25	0.87	Down	TCM_039756	ACACA	Biotin carboxylase 1
18601170	6.8	0.2	-5.09	0.84	Down	TCM_017405	Fab2	Stearoyl-acyl-carrier-protein desaturase
18592535	213.7	20.0	-3.42	0.82	Down	TCM_035438	Fab2	Stearoyl-acyl-carrier-protein desaturase
18588657	266.3	22.6	-3.56	0.83	Down	TCM_037945	FATB	Fatty acyl-ACP thioesterases B
18609697	98.8	9.4	-3.40	0.82	Down	TCM_010763	FabG	NAD(P)-binding Rossmann-fold superfamily protein
18612736	283.0	23.8	-3.57	0.83	Down	TCM_002853	FATA	FatA acyl-ACP thioesterase
18599097	66.1	6.2	-3.42	0.81	Down	TCM_024898	Fab1	3-oxoacyl-acyl-carrier-protein reductase 4
18599592	4.7	105.0	4.47	0.90	Up	TCM_025554	ADH1_7	Alcohol dehydrogenase-like 5
18600736	1.3	579.8	8.77	1.00	Up	TCM_016858	ALDH	Aldehyde dehydrogenase 2B4
18610847	0.1	4.9	6.04	0.84	Up	TCM_000291	EPHX2	Hydrolases superfamily protein
18590027	1.3	20.7	4.02	0.84	Up	TCM_040981	EPHX2	Hydrolases superfamily protein
18591721	2.8	94.4	5.10	0.93	Up	TCM_034407	OPR	12-oxophytodienoate reductase 2
18589945	99.9	6.9	-3.86	0.85	Down	TCM_040854	BAMT	Benzoate carboxyl methyltransferase
18589726	97.6	9.7	-3.32	0.82	Down	TCM_040462	LOX1_5	Linoleate 13S-lipoxygenase 2
18610880	391.6	0.8	-9.03	1.00	Down	TCM_000326	LOX2S	Salicylate O-methyltransferase

Table S4 Differentially expressed fatty acid synthesis-related genes of cacao accession TAS57.

Gene ID	105 DAP	126 DAP	log_2 Fold Change	P-value	Regulated	Name	Gene symbols	Annotation
18588970	11.0	0.6	-4.19	0.84	Down	TCM_038359	KCS	3-ketoacyl-CoA synthase
18601999	5542.6	753.6	-2.88	0.83	Down	TCM_019372	ADH1_7	Lipid-transfer protein
18600500	27.5	2.5	-3.46	0.83	Down	TCM_026683	EPHX2	Hydrolases superfamily protein
18608685	3.3	29.6	3.17	0.82	Up	TCM_008072	EPHX2	Hydrolases superfamily protein
18585925	1.2	13.2	3.48	0.80	Up	TCM_042580		Methyltransferases superfamily protein
18610880	40.5	368.4	3.19	0.85	Up	TCM_000326	LOX2S	Salicylate O-methyltransferase
**Gene ID**	**126 DAP**	**147 DAP**	**log_2 Fold Change**	**P-value**	**Regulated**	**Name**	**Gene symbols**	**Annotation**
18600736	1.0	86.6	6.39	0.97	Up	TCM_016858	FATA	FatA acyl-ACP thioesterase
18585926	13.2	131.3	3.31	0.82	Up	TCM_042582		Methyltransferases superfamily protein
**Gene ID**	**147 DAP**	**168 DAP**	**log_2 Fold Change**	**P-value**	**Regulated**	**Name**	**Gene symbols**	**Annotation**
18608685	18.2	0.5	-5.24	0.89	Down	TCM_008072	EPHX2	Hydrolases superfamily protein
18610880	257.5	1.0	-8.07	0.99	Down	TCM_000326	LOX2S	Salicylate O-methyltransferase
18611607	61.7	6.4	-3.28	0.80	Down	TCM_046682	FabZ	Thioesterase superfamily protein
18592535	121.6	4.1	-4.90	0.92	Down	TCM_035438	Fab2	Stearoyl-acyl-carrier-protein desaturase
18606735	4.7	0.0	-8.88	0.82	Down	TCM_016498	FabF	3-ketoacyl-acyl carrier protein synthase I
18612736	257.7	24.5	-3.40	0.82	Down	TCM_002853	FATA	FatA acyl-ACP thioesterase
18599097	57.5	4.9	-3.55	0.81	Down	TCM_024898	FabI	NAD(P)-binding Rossmann-fold superfamily protein
18586907	27.8	1.8	-3.98	0.83	Down	TCM_044452	PECR	3-oxo-5-alpha-steroid 4-dehydrogenase
18591721	18.7	172.3	3.21	0.81	Up	TCM_034407	OPR	12-oxophytodienoate reductase 2
18589945	71.9	4.1	-4.14	0.86	Down	TCM_040854	BAMT	Benzoate carboxyl methyltransferase

18589726	116.7	4.8	-4.60	0.90	Down	TCM_040462	LOX2S	Linoleate 13S-lipoxygenase 2
18610882	16.1	0.1	-8.07	0.94	Down	TCM_000328	BAMT	Benzoate carboxyl methyltransferase
18608686	154.3	5.1	-4.92	0.92	Down	TCM_008073	EPHX2	Hydrolases superfamily protein
18610847	0.3	8.8	4.90	0.82	Up	TCM_000291	EPHX2	Hydrolases superfamily protein
18600369	3.1	351.8	6.84	0.99	Up	TCM_026544	LOX1_5	Linoleate 9S-lipoxygenase 6
18600366	6.3	1264.9	7.64	1.00	Up	TCM_026539	LOX1_5	Lipoxygenase family protein
18600368	80.1	15558.6	7.60	1.00	Up	TCM_026543	LOX1_5	Linoleate 9S-lipoxygenase 5

Table S5 Differentially expressed TAG synthesis-related genes between cacao accessions TAS42 and TAS57.

Gene ID	TAS57 105 DAP	TAS42 105 DAP	log_2 Fold Change	P-value	Regulated	Name	Gene symbols	Annotation
18604975	12.8	1.0	-3.63	0.80	Down	TCM_014488	ADPRM	ADP-ribose/CDP-alcohol diphosphatase
18603880	7.8	73.8	3.24	0.83	Up	TCM_012037	ALDH	Aldehyde dehydrogenase
Gene ID	TAS57 147 DAP	TAS42 147 DAP	log_2 Fold Change	P-value	Regulated	Name	Gene symbols	Annotation
18588216	53.0	0.01	-12.37	0.98	Down	TCM_037460	PDAT	Phospholipid:diacylglycerol acyltransferase
18597445	34.9	2.9	-3.61	0.81	Down	TCM_021586	AKR1B	NAD(P)-linked oxidoreductase
18589337	8.2	0.1	-6.23	0.87	Down	TCM_039309	ZNF3	Zinc finger protein 3
18611047	0.1	7.5	6.63	0.87	Up	TCM_000525	PDAT	GDSL esterase/lipase
18600884	26.9	0.6	-5.61	0.93	Down	TCM_017024	GPDH	Glycerol-3-phosphate dehydrogenase (NAD+)

Table S6 Differentially expressed TAG synthesis-related genes of cacao accession TAS42.

Gene ID	105 DAP	126 DAP	log_2 Fold Change	P-value	Regulated	Name	Gene symbols	Annotation
18588216	0.2	10.7	5.78	0.90	Up	TCM_037460	PDAT	Phospholipid/diacylglycerol acyltransferase
18609038	14.1	1.0	-3.75	0.80	Down	TCM_008886	PDAT	Phospholipid/diacylglycerol acyltransferase
18604331	34.0	3.2	-3.41	0.82	Down	TCM_012768	PMT	Phosphoethanolamine N-methyltransferase
Gene ID	126 DAP	147 DAP	log_2 Fold Change	P-value	Regulated	Name	Gene symbols	Annotation
18588216	10.7	0.0	-10.07	0.93	Down	TCM_037460	PDAT	Phospholipid/diacylglycerol acyltransferase
18608303	0.2	124.1	8.98	0.99	Up	TCM_007496	PDAT	Phospholipid/diacylglycerol acyltransferase
18596754	3.3	0.0	-8.38	0.81	Down	TCM_029770	PDAT	Phospholipid/diacylglycerol acyltransferase
18606823	0.3	12.7	5.41	0.90	Up	TCM_016600	GPAT	Glycerol-3-phosphate O-acyltransferase
18599648	1.1	17.0	3.97	0.82	Up	TCM_025639	LPAT	Lysophosphatidic acid acyltransferase
18599099	529.1	4917.7	3.22	0.82	Up	TCM_024900	LEA	Late Embryogenesis Abundant
18597445	30.5	2.9	-3.42	0.80	Down	TCM_021586	AKR1B	NAD(P)-linked oxidoreductase
18592835	33.4	1.5	-4.46	0.88	Down	TCM_036186	ZNF	Zinc finger protein
18589337	34.5	0.1	-8.29	0.98	Down	TCM_039309	ZNF3	Zinc finger protein 3
18598114	7.3	0.3	-4.57	0.81	Down	TCM_022373	PDAT	Phospholipid/diacylglycerol acyltransferase
18611047	0.1	7.5	6.63	0.89	Up	TCM_000525	PDAT	GDSL esterase/lipase
18600736	1.3	579.8	8.77	1.00	Up	TCM_016858	ALDH	Aldehyde dehydrogenase 2B4
18600884	57.4	0.6	-6.71	0.97	Down	TCM_017024	GPDH	Glycerol-3-phosphate dehydrogenase (NAD+)
18586972	51.01	1.19	-5.42	0.94	Down	TCM_044588	WRI1	Integrase-type DNA-binding superfamily
Gene ID	147 DAP	168 DAP	log_2 Fold Change	P-value	Regulated	Name	Gene symbols	Annotation
18588761	1.1	22.1	4.30	0.81	Up	TCM_038080	GDE1	SPX domain gene 2

Table S7 Differentially expressed TAG synthesis-related genes of cacao accession TAS57.

Gene ID	105 DAP	126 DAP	log_2 Fold Change	P-value	Regulated	Name	Gene symbols	Annotation
18588216	3.6	27.7	2.96	0.80	Up	TCM_037460	PDAT	Phospholipid/diacylglycerol acyltransferase
18599099	49.2	391.9	2.99	0.83	Up	TCM_024900	LEA	Late Embryogenesis Abundant
18609038	23.9	2.0	-3.57	0.83	Down	TCM_008886	PDAT	Phospholipid/diacylglycerol acyltransferase
18614645	232.4	1358.6	2.55	0.82	Up	TCM_005905	LPAT	Lysophosphatidic acid acyltransferase
Gene ID	126 DAP	147 DAP	log_2 Fold Change	P-value	Regulated	Name	Gene symbols	Annotation
18608303	0.8	21.1	4.73	0.87	Up	TCM_007496	PDAT	Phospholipid/diacylglycerol acyltransferase
18600736	1.0	86.6	6.39	0.97	Up	TCM_016858	ALDH	Aldehyde dehydrogenase 2B4
18604331	12.5	0.8	-4.04	0.80	Down	TCM_012768	PMT	Phosphoethanolamine N-methyltransferase
Gene ID	147 DAP	168 DAP	log_2 Fold Change	P-value	Regulated	Name	Gene symbols	Annotation
18588216	53.0	0.1	-9.79	0.98	Down	TCM_037460	PDAT	Phospholipid/diacylglycerol acyltransferase
18607678	52.9	4.6	-3.53	0.81	Down	TCM_006770	ALDH	Aldehyde dehydrogenase
18598114	6.3	0.1	-5.59	0.82	Down	TCM_022373	PDAT	Phospholipid/diacylglycerol acyltransferase
18611047	0.1	7.7	6.68	0.87	Up	TCM_000525	PDAT	GDSL esterase/lipase
18601777	20.9	1.5	-3.85	0.81	Down	TCM_019014		Uncharacterized protein
18600884	26.9	0.4	-6.11	0.94	Down	TCM_017024	GPDH	Glycerol-3-phosphate dehydrogenase (NAD+)
18586972	51.01	1.19	-5.42	0.94	Down	TCM_044588	WRI1	Integrase-type DNA-binding superfamily

Table S8 Primer sequences used in this study.

Gene IDs	Gene symbols	Forwards primers	Reverse primers
CGD0021790	Actin	TCCTCTTCCAGCCATCTCTC	TCTCCTTGCTCATTCGGTCT
18592535	Fab2	CAAACACCCACTGCCTTTC	CAAAAAAGTCTGGGGTTGCC
18601170	Fab2	CATCATTATTCCGCTGTTGC	TAGAGGGCAGTCCGCAAAC
18606735	FabF	GCAACTGCTGCTTGTCCTT	CCACAGCCCTGATTGTTCT
18612736	FATA	ACAGGATGACGTGGTGGAT	CTCAGTACGGCCTCGATTT
18588970	KCS	CTGTACTGGACGAGTTGGAG	CTCCTTAGCTGGATTTATGGT
18601999	ADH1_7	CACGGTGGAGGCAGTGAA	GGTATGTCACGCCGCAAG
18600736	ALDH	GCCAACACCTTGACCCGT	CAACCACGCTGGATGCTT
18588216	PDAT	TTGTCGACTACCTCCAGGTGT	GAGAATCGCCCTGTTGGTT
18596754	PDAT	TGGCAACCTTGAGGACAAC	ATGAACAGCATCCCAGAATAC
18597445	AKR1B	CTGGTCAGGTGCTGGTAAA	CCTAAAGAGCCCAACATCC
18599099	LEA	AACAGGCACCCATACTTACA	GGGTATTATGGGTCTTGGAC
18606823	GPAT	CATTCTTCAGCGGCACTTC	GTACGGGAATGCTCAACCT
18591721	OPR	TAGAGCCGAGGATGGTCAC	ACAGGATCATGCGTGTAAAAG
18589726	LOX2S	CAAAACTTTATTCCCACTTCG	AGCCCTCGGTCTAGCCTTA
18608685	EPHX2	GGTTCCTTACAGGTGCTC	GTTCATAAACCAGCTTCATC
